# Atlanta Medical College

**Published:** 1861-05

**Authors:** 


### Atlanta Medical College.

This institution, which is now entering upon its seventh regular course, with a steady increase in the number of students every year, opens with a class much larger than was expected in the present disturbed state of the country. The session commences with a larger number of students than usual, and, but for the large number of medical students whose noble hearts are moved with patriotism to volunteer in the defense of our country, the class would have been double the size of any former one. While we feel an admiration of the motive which actuates those who deny themselves the benefits of the present course here, and while the Faculty feel intensely the emotions which pervade Southern hearts, they determine to conduct the usual exercises of the College, regularly through the course. The full amount of lectures in every department will be given, with the full amount of dissections, demonstrations and clinical instruction.

Some members of the Faculty have with difficulty been induced to remain during the course, and a majority of its members will doubtless go into active military service, so soon as the session closes.

From recent demonstrations we are thoroughly satisfied that no class in our community exhibits more determined zeal in the defense of our government, than those who have selected the profession of medicine.

